# Model of Break-Bone Fever via Beta-Derivatives

**DOI:** 10.1155/2014/523159

**Published:** 2014-09-11

**Authors:** Abdon Atangana, Suares Clovis Oukouomi Noutchie

**Affiliations:** ^1^Institute for Groundwater Studies, Faculty of Natural and Agricultural Sciences, University of the Free State, Bloemfontein 9300, South Africa; ^2^Ma SIM Focus Area, North-West University, Mafikeng 2735, South Africa

## Abstract

Using the new derivative called beta-derivative, we modelled the well-known infectious disease called break-bone fever or the dengue fever. We presented the endemic equilibrium points under certain conditions of the physical parameters included in the model. We made use of an iteration method to solve the extended model. To show the efficiency of the method used, we have presented in detail the stability and the convergence of the method for solving the system (2). We presented the uniqueness of the special solution of system (2) and finally the numerical simulations were presented for various values of beta.

## 1. Introduction

In the last two centuries, several new infectious diseases have been discovered. Their mode of transmission differs from one disease to another. In some cases the transmission is in direct contact with the patient; see, for instance, HIV. The transmission can also take place in air, for instance, TB. In other cases the transmission is indirect; the virus is transported by a vector such as a mosquito and others. One of these infectious diseases is the so-called dengue fever also known as break-bone fever. The first record of this infectious disease can be traced back in a Chinese medical instruction book from the Jin Dynasty (265–420° AD) which referred to a water poison associated with flying insects [1, 2]. The primary vector,* A. aegypti*, extended to Africa in the 15th to 19th centuries because of the increased globalization secondary to the slave trade [3]. In many years to follow, there have been metaphors of epidemics in the 17th century, other than the most credible premature reports of dengue epidemic are from 1779 and 1780, when an epidemic brushed away crosswise Asia, Africa, and North America [2].

This disease is transmitted by several species of mosquito within the genus* Aedes*, principally* A. aegypti*. The virus has five different types; infection with one type usually gives lifelong immunity to that type, but only short term immunity to the others [4]. When a mosquito carrying dengue virus bites a person, the virus enters the skin together with the mosquito saliva. It attaches to and enters white blood cells and duplicates inside the cells at the same time as they progress all the way through the body. In the process of defense, the white blood cells take action by producing a number of signalling proteins, such as cytokines and interferons, which are responsible for many symptoms. This mechanism can be converted in mathematical equations.

SEIR model is one mathematical equation underpinning the analysis of the simulation of the spread of dengue virus between host and vector. A well-established knowledge regarding the mathematical formulation of the model for the human and mosquito populations can be found in [5] and is given as

(1) $$\begin{array}{c} \frac{dS_{h}}{dt}=\mu_{h}N_{h}-\left(\frac{\beta_{h}bI_{v}}{N_{h}}+p+\mu_{h}\right)S_{h},\\ \frac{dE_{h}}{dt}=\left(\frac{\beta_{h}bI_{v}}{N_{h}}+p\right)S_{h}-\left(\mu_{h}+\delta_{h}\right)E_{h},\\ \frac{dI_{h}}{dt}=\varphi_{h}E_{h}-\left(\mu_{h}+\gamma_{h}+\alpha_{h}\right)I_{h},\\ \frac{dE_{v}}{dt}=\frac{\beta_{v}bI_{h}}{N_{h}}\left(\frac{A}{\mu_{v}}-E_{v}-I_{v}\right)-\left(\mu_{v}+\delta_{v}\right)E_{v},\\ \frac{dI_{v}}{dt}=\delta_{v}E_{v}-\mu_{v}I_{v}, \end{array}$$

where *N*_*h*_ is the host population, *μ*_*h*_ and *μ*_*v*_ are the death rate of host and vector populations, respectively, *β*_*h*_ and *β*_*v*_ are the transmission probability from vector to host and from host to vector, respectively, *b* is the biting rate of the vector, *I*_*v*_ and *I*_*h*_ are infected vector and host population, respectively, *S*_*h*_ is the number of susceptible persons in the host population, *A* is the recruitment rate of the vector host, *γ*_*h*_ is the recovery rate of the host population, *δ*_*v*_ is the proportional rate of the mosquitoes exposed to the virus infection, and *α*_*h*_ is the rate of death caused by dengue fever. In the recent years scholars in the area of applications of ordinary and partial differential equations have paid their attentions to investigate which concept of derivative is suitable for modeling real world problems [5, 6]. The outcome of these investigations revealed that it is more suitable to model real world problems with derivative based on the fractional concept than the classical version. The derivative based on the concept of fractional order has therefore gained the world of modeling in the recent decade including in the field of hydrology studies, chemistry, engineering, and mathematical biology [7–12]. With the rewards of fractional derivatives, several new definitions have been introduced recently [13, 14]. In the same line of idea, we have put in place a new derivative called the *β*-derivative; this derivative may not be seen as fractional derivative but has fractional compound [15, 16]. We have used this derivative in our previous work and the results obtained were very interesting. Therefore in this work our main interest is to extend (1) using the new derivative; a stability analysis will be presented and finally a special solution using some interesting iterations methods will be presented as well. The extended version of (1) is given by

(2) Dtβ0ASh=μhNh−(βhbIvNh+p+μh)Sh,Dtβ0AEh=(βhbIvNh+p)Sh−(μh+δh)Eh,Dtβ0AIh=φhEh−(μh+γh+αh)Ih,Dtβ0AEv=βvbIhNh((Aμv−Ev−Iv))−(μv+δv)Ev,Dtβ0AIv=δvEv−μvIv,

where

(3) D0Axβ(f(x))=lim⁡ε→0⁡f(x+ε(x+(1/Γ(β)))1−β)−f(x)ε

for all *x* ≥ *a*, *β* ∈ (0,1]. When the limit of the above exists, *f* is said to be *β*-differentiable.

**Theorem 1 (see [16]).**

Assuming that *f* is differential and *β*-differentiable on the opened interval (*a*, *b*), then

(4) D0Axβ(f(x))=(x+1Γ(β))1−βlim⁡h→0⁡f(x+h)−f(x)h.

**Definition 2 (see [16]).**

Let *f* : [*a*, *∞*) → *R* be a given function; then we propose that the integral of order *β*-integral of *f* is

(5) IaAxβ(f(x))=∫ax(t+1Γ(β))β−1f(t)dt.

The above operator is the inverse operator of the proposed beta-derivative and is called the Atangana ‘beta integral.

## 2. Endemic Equilibrium

In this section, we will present the endemic equilibrium points and also present the stability analysis. If we assume that the system of equations does not depend on time, beta-derivative allows us to have

(6) $$\begin{array}{c} 0=\mu_{h}N_{h}-\left(\frac{\beta_{h}b{\overset{-}{I}}_{v}}{N_{h}}+p+\mu_{h}\right){\overset{-}{S}}_{h},\\ 0=\left(\frac{\beta_{h}b{\overset{-}{I}}_{v}}{N_{h}}+p\right){\overset{-}{S}}_{h}-\left(\mu_{h}+\delta_{h}\right){\overset{-}{E}}_{h},\\ 0=\varphi_{h}{\overset{-}{E}}_{h}-\left(\mu_{h}+\gamma_{h}+\alpha_{h}\right){\overset{-}{I}}_{h},\\ 0=\frac{\beta_{v}b{\overset{-}{I}}_{h}}{N_{h}}\left(\frac{A}{\mu_{v}}-{\overset{-}{E}}_{v}-{\overset{-}{I}}_{v}\right)-\left(\mu_{v}+\delta_{v}\right){\overset{-}{E}}_{v},\\ 0=\delta_{v}{\overset{-}{E}}_{v}-\mu_{v}{\overset{-}{I}}_{v}. \end{array}$$

It is worth noting that there is no general solution of the above equation in the literature; therefore in this world we will provide a general solution of the above system under some condition on the physical parameters. Consider

(7) E−v=μvI−vδv,  I−h=(μv+δv)(μv/δv)·(Nh/βvb)(A/μvI−v)−(μv/δv)−1,E−h=μh+γh+αhρh(μv+δv)(μv/δv)·(Nh/βvb)(A/μvI−v)−(μv/δv)−1,S−h=μv+δv(bβhI−v/Nh)+p ×μh+γh+αhρh(μv+δv)(μv/δv)·(Nh/βvb)(A/μvI−v)−(μv/δv)−1,S−h=μv+δv(bβhI−v/Nh)+p+μh.

However, to find ${\overset{-}{I}}_{v}$ we will solve the following equation:

(8) μv+δv(bβhI−v/Nh)+pμh+γh+αhρh(μv+δv)(μv/δv)·(Nh/βvb)(A/μvI−v)−(μv/δv)−1 =μv+δv(bβhI−v/Nh)+p+μh.

Nonetheless, for simplicity, we put

(9) $$\begin{array}{c} \frac{\mu_{h}+\gamma_{h}+\alpha_{h}}{\rho_{h}}=a_{h},\;\;a_{1}=\left(\mu_{v}+\delta_{v}\right)\frac{\mu_{v}}{\delta_{v}}\cdot \frac{N_{h}}{\beta_{v}b},\\ a_{2}=\frac{A}{\mu_{b}},\;\;a_{3}=\frac{b\beta_{h}}{N_{h}},\;\;a_{4}=\frac{p}{N_{h}},\\ a_{5}=\mu_{h}+p,\;\;a_{6}=\mu_{h}+S_{h},\\ a_{7}=\mu_{h}+\delta_{h},\;\;x={\overset{-}{I}}_{v}. \end{array}$$

Then (8) can be converted to

(10) $$\begin{array}{c} \frac{a_{7}}{a_{3}x+a_{5}}=\frac{a_{6}a_{h}}{a_{3}x+a_{4}}\cdot \frac{a_{1}x}{a_{2}-a_{4}x},\\ Rx^{2}+Bx-C=0\\ R=a_{7}a_{3}a_{4}+a_{4}^{2}+a_{h}a_{6}a_{1}a_{3},\\ B=a_{h}a_{6}a_{1}a_{3}-a_{7}a_{3}a_{4}-a_{7}a_{2}a_{4},\;\;C=a_{7}a_{2}a_{4}. \end{array}$$

The solution of (10) is given as

(11) $$\begin{array}{c} x_{\mp }=\frac{-B\mp \sqrt{B^{2}-4RC}}{2R}. \end{array}$$

Now according to the physical meaning of our problem, we chose only the positive solution and we have the last equilibrium endemic point ${\overset{-}{I}}_{v}$. The endemic equilibrium points are given as

(12) I−v=((μh+δh)AμbpNh+(μh+δh)pNhbβhNh  −(μv+δv)μvδv·Nhβvb(μh+Sh)bβhNh(μh+γh+αhρh)) ×(2{(μv+δv)μvδv·Nhβvb(μh+Sh)     ×bβhNh(μh+γh+αhρh)+(pNh)2     +(μh+δh)pNhbβhNh})−1 +{((μh+δh)AμbpNh+(μh+δh)pNhbβhNh   −(μv+δv)μvδv·Nhβvb(μh+Sh)bβhNh(μh+γh+αhρh))2   −4({(μv+δv)μvδv·Nhβvb(μh+Sh)      ×bβhNh(μh+γh+αhρh)+(pNh)2      +(μh+δh)pNhbβhNh})(μh+δh)AμbpNh    +(μh+δh)pNhbβhNh}1/2,E−v=μvI−vδv,  I−h=(μv+δv)(μv/δv)·(Nh/βvb)(A/μvI−v)−(μv/δv)−1,E−h=μh+γh+αhρh(μv+δv)(μv/δv)·(Nh/βvb)(A/μvI−v)−(μv/δv)−1.

## 3. Method for Solving the System

One of the important aspects in modeling is not only to formulate the physical problem into a mathematical equation, but also to be able to predict the behaviour of this physical problem. This can only be achieved by finding the solution of the system. The problem under investigation is a nonlinear problem and needs an efficient analytical technique to derive a special solution of the system. In this paper we will use the so-called homotopy decomposition method to achieve this. The methodology of this technique can be found in several papers, for instance, in [17, 18]. But in this paper, we will only apply the method to solve the system (2). Therefore applying the method on system (2), we obtain the following iteration formulas:

(13) Sh0(t)=Sh(0),Eh0(t)=Eh(0),Ih0(t)=Ih(0),Ev0(t)=Ev(0),Iv0(t)=Iv(0),Sh1=I0Atβ(μhNh−(βhbIv0Nh+p+μh)Sh0),Eh1=I0Atβ((βhbIv0Nh+p)Sh0−(μh+δh)Eh0),Ih1=I0Atβ(φhEh0−(μh+γh+αh)Ih0),Ev1=I0Atβ(βvbIh0Nh((Aμv−Ev0−Iv0))    −(μv+δv)Ev0),Iv1=I0Atβ(δvEv0−μvIv0),Sh(t)=I0Atβ(μhNh−(βhbGv(n−1)Nh)+(p+μh)Sh(n−1))Eh(t)=I0Atβ((βhbNh)Gv(n−1)+pSh(n−1)−(μh+δh)Eh(n−1))Ih(t)=I0Atβ(φhEh(n−1)−(μh+γh+αh)Ih(n−1))Ev(t)=I0Atβ(βvbNh((Aμv−Tv(n−1)−Kv(n−1)))      −(μv+δv)Ev(n−1))Iv(n)(t)=I0Atβ(δvEv(n−1)−μvIv(n−1)),

where

(14) Gv(n−1)(t)=∑j=0n−1Iv(j)Sh(n−1−j),Tv(n−1)(t)=∑j=0n−1Iv(j)Eh(n−1−j),Kv(n−1)(t)=∑j=0n−1Iv(j)Iv(n−1−j).

### 3.1. Stability Analysis

Before the presentation of the stability, we will first present the following operator, which will be referred to as Atangana ‘beta inner product.

**Definition 3 .**

A function *f* defined on [*a* *b*] is said to be beta-integrable if

(15) $$\begin{array}{c} \overset{b}{\underset{0}{\int }}{\left(t+\frac{1}{\Gamma \left(\beta \right)}\right)}^{\beta -1}f\left(t\right)dt \end{array}$$

exists.

**Definition 4 .**

Let *f* and *g* be two functions defined on [0, *b*]. Assuming that *fg* is beta-integrable, then the beta inner product is defined as

(16) $$\begin{array}{c} A\left(f,g\right)=\overset{b}{\underset{0}{\int }}{\left(t+\frac{1}{\Gamma \left(\beta \right)}\right)}^{\beta -1}f\left(t\right)g\left(t\right)dt. \end{array}$$

We will present some properties of the above operator.

*Properties*

1. *A*(*f*, *g*) = *A*(*g*, *f*): the operator is symmetric;
2. *A*(*f*, *ag* + *bh*) = *aA*(*f*, *g*) + *bA*(*f*, *h*), any constant in real space;
3. *A*(*f*, *g*) = 0 if *g* = 0 or *f* = 0;
4. *A*(*f*, *f*) > 0 if *f* ≠ 0;
5. if *f* and *g* are bounded and are positive functions in [0   *b*], then *A*(*f*, *g*) is bounded in [0   *b*].

**Proof**

Consider

(17) A(f,g)=∫0b(t+1Γ(β))β−1f(t)g(t)dt=∫0b(t+1Γ(β))β−1g(t)f(t)dt=A(g,f)A(f,ag+bh)=∫0b(t+1Γ(β))β−1f(t)(ag+bh)dt=a∫0b(t+1Γ(β))β−1g(t)f(t)dt +b∫0b(t+1Γ(β))β−1h(t)f(t)dt=aA(f,g)+bA(f,h).

*A*(*f*, *g*) = 0 implies that ∫_0_^*b*^(*t*+(1/Γ(*β*)))^*β*−1^ *f*(*t*)*g*(*t*)*dt* = 0; using the integral properties, we obtain (*t*+(1/Γ(*β*)))^*β*−1^ *f*(*t*)*g*(*t*) = 0 for all *t* in [*a* *b*]; then *f*(*t*)*g*(*t*) = 0 for all *t* in [0   *b*] since (*t*+(1/Γ(*β*)))^*β*−1^ ≠ 0; thus, *f*(*t*) = 0 or *g*(*t*) = 0 for all *t* in [0   *b*]:

(18) $$\begin{array}{c} A\left(f,f\right)=\overset{b}{\underset{0}{\int }}{\left(t+\frac{1}{\Gamma \left(\beta \right)}\right)}^{\beta -1}{f\left(t\right)}^{2}dt. \end{array}$$

However, for all *t* in [0  *b*], (*t*+(1/Γ(*β*)))^*β*−1^ *f*(*t*)^2^ > 0 by applying integral sign we obtain

(19) $$\begin{array}{c} \overset{b}{\underset{0}{\int }}{\left(t+\frac{1}{\Gamma \left(\beta \right)}\right)}^{\beta -1}{f(t)}^{2}dt>0⟹A\left(f,f\right)>0. \end{array}$$

Assume that *f* and *g* are bounded in [0  *b*]; then we can find two real numbers, say *F* and *M*, such that for all *t* in [0  *b*]*f*(*t*) < *M* and *g*(*t*) < *F*; this implies *f*(*t*)*g*(*t*) < MF; thus

(20) A(f,g)=∫0b(t+1Γ(β))β−1f(t)g(t)dt<MF∫0b(t+1Γ(β))β−1dt=MFPP=(b+(1/Γ(β)))β−(a+(1/Γ(β)))ββ.

With the above information in hand, we will now prove the stability of the method for solving the system (2). To achieve this, we will in addition consider the following operator:

(21) T(u,v,w,s,z)={μhNh−(βhbsNh+p+μh)u,(βhbsNh+p)u−(μh+δh)v,φhv−(μh+γh+αh)w,βvbsNh((Aμv−z−s))−(μv+δv)z,δvz−μvs.

**Theorem 5 .**

Let us consider the operator *T* and consider the initial and boundary condition for (2); then the new variation iteration method leads to a special solution of (2).

**Proof**

To achieve this we will think about the following *f* sub-Hilbert space of the Hilbert space *H* = *L* ^2^((0, *T*)) [13] that can be defined as the set of those functions in the following space:

(22) $$\begin{array}{c} v:\left(0,T\right)⟶R,\;\;B=\left\{u,v\;|\;A\left(u,v\right)<\infty \right\}. \end{array}$$

We harmoniously assume that the differential operators are restricted under the *L* ^2^ norms. Using the definition of the operator *T* we have the following:

(23) T(u,v,w,s,z)−T(u1,v1,w1,s1,z1)={−(p+μh)(u−u1)−βhbNh(us−u1s1),(βhbNh)(su−s1u1)+p(u−u1)  −(μh+δh)(v−v1),φh(v−v1)−(μh+γh+αh)(w−w1),−βhbNh((z−z1)+(s−s1))−(μv+δv)(z−z1)  +Aμvβvb(s−s1)Nhδv(z−z1)−μv(s−s1).

We will now evaluate the inner product of *O* = (*T*(*u*, *v*, *w*, *s*, *z*) − *T*(*u*_1_, *v*_1_, *w*_1_, *s*_1_, *z*_1_), (*u* − *u*_1_, *v* − *v*_1_, *w* − *w*_1_, *s* − *s*_1_, *z* − *z*_1_)):

(24) O={(−(p+μh)(u−u1)−βhbNh(us−u1s1),u−u1),((βhbNh)(su−s1u1)+p(u−u1) −(μh+δh)(v−v1),v−v1),(φh(v−v1)−(μh+γh+αh)(w−w1),w−w1),(−βhbNh((z−z1)+(s−s1))−(μv+δv)(z−z1) +Aμvβvb(s−s1)Nh,s−s1),(δv(z−z1)−μv(s−s1),z−z1).

We will evaluate the above row after row. Now using the properties of the inner function, we obtained the following:

(25) (−(p+μh)(u−u1)−βhbNh(us−u1s1),u−u1) ≤||−u+u1||{(p+μh)||−u+u1||+||βhbNh(us−u1s1)||} =k1||u−u1||((βhbNh)(su−s1u1)+p(u−u1) −(μh+δh)(v−v1),v−v1) ≤||v−v1||(||v−v1||(μh+δh)+p||u−u1||        +||(βhbNh)(su−s1u1)||)=k2||v−v1||(φh(v−v1)−(μh+γh+αh)(w−w1),w−w1) ≤||w−w1||{||−w+w1||(μh+γh+αh)+||φh(v−v1)||} =k3||w−w1||(−βhbNh((z−z1)+(s−s1))−(μv+δv)(z−z1) +Aμvβvb(s−s1)Nh,s−s1) ≤||s−s1||{||Aμvβvb(s−s1)Nh||+(μv+δv)||z−z1||       +βhbNh(||−z+z1||+||−s+s1||)} =k4||s−s1||(δv(z−z1)−μv(s−s1),z−z1) ≤||z−z1||(||δv(z−z1)||+μv||−s+s1||)=k5||z−z1||.

Therefore, we have that

(26) O≤{k1||u−u1||,k2||v−v1||,k3||w−w1||,k4||s−s1||,k5||z−z1||.

It follows that it is possible to find a positive *K*(*k*_1_, *k*_2_, *k*_3_, *k*_4_, *k*_5_) such that

(27) (T(u,v,w,s,z)−T(u1,v1,w1,s1,z1), (u−u1,v−v1,w−w1,s−s1,z−z1  ))≤K||V−V1||,

with *V* = (*u*, *v*, *w*, *s*, *z*) and *V*_1_ = (*u*_1_, *v*_1_, *w*_1_, *s*_1_, *z*_1_). We will prove that we can also find a positive constant *P* = (*p*_1_, *p*_2_, *p*_3_, *p*_4_, *p*_5_) such that for all  *Q* = (*q*_1_, *q*_2_, *q*_3_, *q*_4_, *q*_5_)

(28) O1=(T(u,v,w,s,z)  −T(u1,v1,w1,s1,z1),(q1,q2,q3,q4,q5))≤{p1||u−u1||||q1||,p2||v−v1||||q2||,p3||w−w1||||q3||,p4||s−s1||||q4||,p5||z−z1||||q5||.

In fact,

(29) O1={(−(p+μh)(u−u1)−βhbNh(us−u1s1),q1  )((βhbNh)(su−s1u1)+p(u−u1) −(μh+δh)(v−v1),q2)(φh(v−v1)−(μh+γh+αh)(w−w1),q3)(−βhbNh((z−z1)+(s−s1)) −(μv+δv)(z−z1)+Aμvβvb(s−s1)Nh,q4)(δv(z−z1)−μv(s−s1),q5).

Again, using a similar method that we used earlier, we obtain the following inequality:

(30) $$\begin{array}{c} O_{1}\leq \{\begin{array}{cc} p_{1}||u-u_{1}||||q_{1}||, & \\ p_{2}||v-v_{1}||||q_{2}||, & \\ p_{3}||w-w_{1}||||q_{3}||, & \\ p_{4}||s-s_{1}||||q_{4}||, & \\ p_{5}||z-z_{1}||||q_{5}||. & \end{array} \end{array}$$

Therefore

(31) (T(u,v,w,s,z)−T(u1,v1,w1,s1,z1), (q1,q2,q3,q4,q5))≤P||V−V1||||Q||.

Inequalities (31) and (27) guaranty the stability of the method used to solve (2) and also lead us to a special solution of (2). We will now show in detail the uniqueness of the special solution.

### 3.2. Uniqueness of the Special Solution

**Theorem 6 .**

The special solution obtained via the used method is unique.

**Proof**

Assuming that *W* is the exact solution of system (2), let *V* and *V*_1_ be two different special solutions of system and converge to *W* ≠ 0 for some large numbers *n* and *m* (2) while using the homotopy method; then using Theorem 5, we have the following inequality:

(32) (T(u,v,w,s,z)−T(u1,v1,w1,s1,z1), (w1,w2,w3,w4,w5))≤P||V−V1||||W||,P||V−V1||||W||≤P||V−W+W−V1||||W||.

Using the triangular inequality, we arrive at the following:

(33) $$\begin{array}{c} P||V-V_{1}||||W||\leq P\left\{||W-V_{1}||+||V-W||\right\}||W||. \end{array}$$

However, since *V* and *V*_1_ converge to *W* for large numbers *n* and *m*, then we can find a small positive parameter *ε*, such that

(34) $$\begin{array}{c} ||W-V_{1}||<\frac{\varepsilon }{2P||W||},\;\text{for}\;n,\\ ||V-W||<\frac{\varepsilon }{2P||W||},\;\text{for}\;m. \end{array}$$

Now consider *M* = max⁡(*n*, *m*); then

(35) P||V−V1||||W||≤P{||W−V1||+||V−W||}||W||<ε2P||W||+ε2P||W||=ε for  M.

Then borrowing the topology idea, we have that

(36) $$\begin{array}{c} P||V-V_{1}||||W||=0. \end{array}$$

Since *W* ≠ 0 and *P* ≠ 0, then ||*V* − *V*_1_|| = 0 implying *V* = *V*_1_. This shows the uniqueness of the special solution.

### 3.3. Algorithm

We will give the following code that will be used to derive the special solution of system (2):

(i) input:
  (37) Sh0(t)=Sh(0)Eh0(t)=Eh(0)Ih0(t)=Ih(0)Ev0(t)=Ev(0)Iv0(t)=Iv(0)
  as preliminary input;
(ii) *j*: number of terms in the rough calculation;
(iii) output:
  (38) Shapp(t),Ehapp(t)Ihapp(t)Evapp(t)Ivapp(t),
  the approximate solution.

**Step 1 .**

Put

(39) $$\begin{array}{c} \{\begin{array}{cc} S_{h0}\left(t\right)=S_{h}\left(0\right) & \\ E_{h0}\left(t\right)=E_{h\;}\left(0\right) & \\ I_{h0}\left(t\right)=I_{h}\;\left(0\right) & \\ E_{v0}\left(t\right)=E_{v}\;\left(0\right) & \\ I_{v0}\left(t\right)=I_{v}\left(0\right), & \end{array}\;\;\{\begin{array}{cc} S_{h\text{app}}\left(t\right) & \\ E_{h\text{app}}\left(t\right) & \\ I_{h\text{app}}\left(t\right) & \\ E_{v\text{app}}\left(t\right) & \\ I_{v\text{app}}\left(t\right) & \end{array}=\{\begin{array}{cc} S_{h0}\left(t\right) & \\ E_{h0}\left(t\right) & \\ I_{h0}\left(t\right) & \\ E_{v0}\left(t\right) & \\ I_{v0}\left(t\right). & \end{array} \end{array}$$

**Step 2 .**

For *j* = 1 to *n* − 1 do Step 3, Step 4, and Step 5:

(40) Sh1=I0Atβ(μhNh−(βhbIv0Nh+p+μh)Sh0),Eh1=I0Atβ((βhbIv0Nh+p)Sh0−(μh+δh)Eh0  ),Ih1=I0Atβ(φhEh0−(μh+γh+αh)Ih0),Ev1=I0Atβ(βvbIh0Nh((Aμv−Ev0−Iv0))−(μv+δv)Ev0),Iv1=I0Atβ(δvEv0−μvIv0).

**Step 3 .**

Compute

(41) Ch(n)(t)=I0Atβ(μhNh−(βhbGv(n−1)Nh)+(p+μh)Sh(n−1)),Hh(n)(t) =I0Atβ((βhbNh)Gv(n−1)+pSh(n−1)−(μh+δh)Eh(n−1))Lh(n)(t)=I0Atβ(φhEh(n−1)−(μh+γh+αh)Ih(n−1))Hv(n)(t)=I0Atβ(βvbNh((Aμv−Tv(n−1)−Kv(n−1)))        −(μv+δv)Ev(n−1))Lv(n)(t)=I0Atβ(δvEv(n−1)−μvIv(n−1)).

**Step 4 .**

Compute

(42) Ch(n+1)(t)=Ch(n)(t)+Sh(app)(t),Eh(n+1)(t)=Eh(n)(t)+Eh(app)(t),Ih(n+1)(t)=Ih(n)(t)+Ih(app)(t),Ev(n+1)(t)=Ev(n)(t)+Ev(app)(t),Iv(n+1)(t)=Iv(n)(t)+Iv(app)(t),Gv(n−1)(t)=∑j=0n−1Iv(j)Sh(n−1−j),Tv(n−1)(t)=∑j=0n−1Iv(j)Eh(n−1−j),Kv(n−1)(t)=∑j=0n−1Iv(j)Iv(n−1−j).

**Step 5 .**

Compute

(43) Shapp(t)Ehapp(t)Ihapp(t)Evapp(t)Ivapp(t)={Shapp(t)+Ch(n+1)(t)Ehapp(t)+Eh(n+1)(t)Ihapp(t)+Ih(n+1)(t)Evapp(t)+Ev(n+1)(t)Ivapp(t)+Iv(n+1)(t).

Stop.

The above algorithm will be used to derive the special solution of system (2).

## 4. Numerical Solution

The above algorithm will be used to produce the numerical solution of system (2) for given values of parameters that can also be found in the literature. We chose the following:

(44) $$\begin{array}{c} \frac{5070822}{5071126}=S_{h}\left(0\right),\\ \frac{50711}{5071126}=E_{h}\left(0\right),\\ \frac{304}{5071126}=I_{h}\left(0\right),\\ 0.01=E_{v}\left(0\right),\\ 0.1=I_{v}\left(0\right). \end{array}$$

Now employing the above algorithm, we obtain

(45) Sh1(t)−(0.004633266482631293     ×((1Gamma[β])−β−(t+1Gamma[β])−β      ×(1+tGamma[β])2))     ×((−2+β)Gamma[β]2)−1,Eh1(t)=(0.8984731674318148     ×((1Gamma[β])−β−(t+1Gamma[β])−β       ×(1+tGamma[β])2))    ×((−2+β)Gamma[β]2)−1,Ih1(t) =(0.00002001739006287755   ×((1Gamma[β])−β    −(t+1Gamma[β])−β(1+tGamma[β])2))  ×((−2+β)Gamma[β]2)−1,Ev1(t) =(0.008558681695478807   ×((1Gamma[β])−β     −(t+1Gamma[β])−β(1+tGamma[β])2))  ×((−2+β)Gamma[β]2)−1,Iv1(t) =(0.0013830000000000003   ×((1Gamma[β])−β    −(t+1Gamma[β])−β(1+tGamma[β])2))  ×((−2+β)Gamma[β]2)−1.

**Figure 1:**
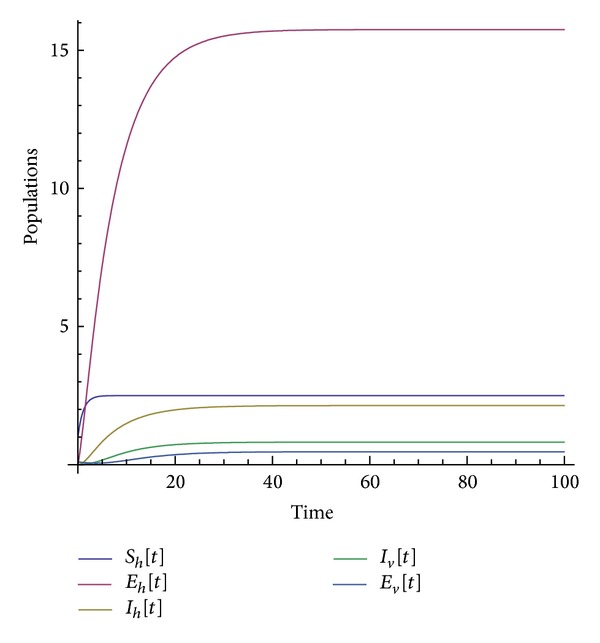
Numerical simulation of the population solution for beta = 1.

**Figure 2:**
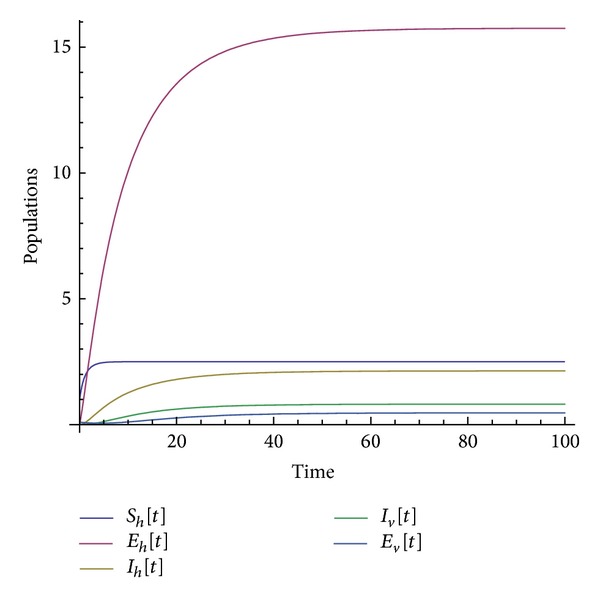
Numerical simulation of the population solution for beta = 0.85.

**Figure 3:**
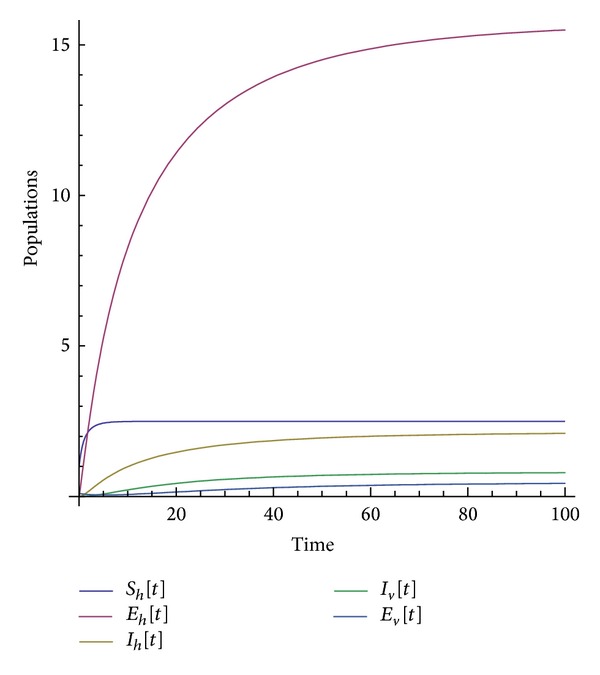
Numerical simulation of the population solution for beta = 0.65.

**Figure 4:**
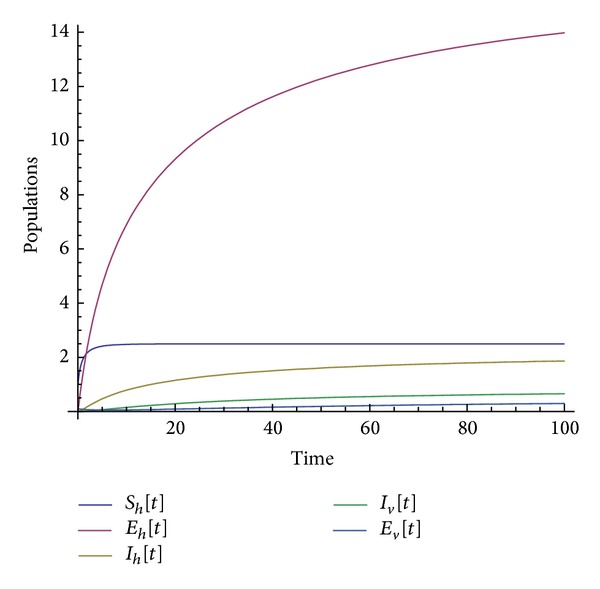
Numerical simulation of the population solution for beta = 0.45.

**Figure 5:**
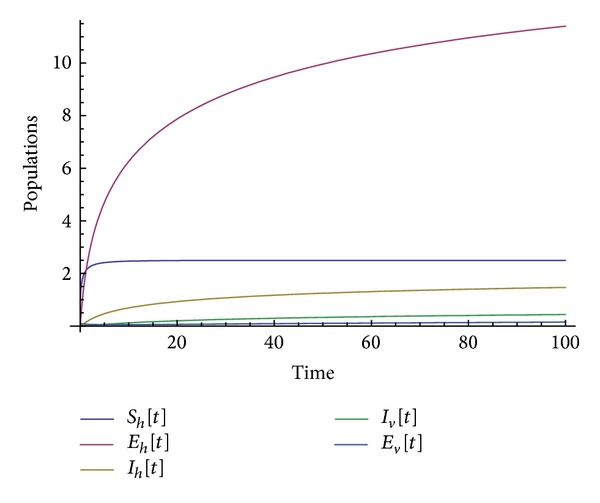
Numerical simulation of the population solution for beta = 0.45.

Many other terms can be computed using the algorithm. The numerical simulations of the special solution for the first two components are depicted in Figures 1, 2, 3, 4, and 5. It is very clear from Figures 3, 4, and 5 that the model depends on the parameter beta; precisely, we observed that the set of solutions is much dependent on the parameter beta; as beta decreases, the set of numerical solutions also decreases.

## 5. Conclusion

In the last decade mathematic tools have been used to model several physical phenomena, for instance, infectious diseases. These mathematical equations describing infectious diseases are using the idea of derivative. Nowadays there exist several derivatives in the literature; all of them have their strength and their weaknesses. For example, the fractional derivative according to Riemann-Liouville and Caputo is not obeying the product, quotient, and chain rule. A new derivative called beta-derivative was used to model the break-bone disease. The resulting system of equations was examined in the scope of an iteration method. For the first time, an analytical expression underpinning the endemic equilibrium points was presented. The efficacy of the used method was demonstrated via the stability and convergence analysis. A relatively new inner product was proposed and was used to prove the uniqueness of the special solution. Numerical simulations were depicted in Figures 1, 2, 3, 4, and 5 for a given value of beta. The derivative used here will shed light on the field of modeling.
